# Non-Dispersive Gas Analyzer for H_2_O and CO_2_ Flux Analysis by the Eddy Covariance Method

**DOI:** 10.3390/s26020560

**Published:** 2026-01-14

**Authors:** Igor Fufurin, Ivan Karpov, Alisa Kosterova, Viacheslav Bessonov, Alexis Yaroslavtsev, Ivan Seregin, Andrey Morozov

**Affiliations:** 1 Physics Department, Bauman Moscow State Technical University, 105005 Moscow, Russia; karpov@bmstu.ru (I.K.); kosterova.a@bmstu.ru (A.K.); bessonov.v@bmstu.ru (V.B.); amor59@mail.ru (A.M.); 2 Ecology Department, Russian State Agrarian University—Moscow Timiryazev Agricultural Academy, 127434 Moscow, Russia; yaroslavtsevam@rgau-msha.ru (A.Y.); iseregin@rgau-msha.ru (I.S.)

**Keywords:** NDIR spectroscopy, gas analyzer, eddy covariance method, flux measurement

## Abstract

This article presents the stages of development of a non-dispersive infrared (NDIR) open-type gas analyzer prototype (NGAP-1) with a fundamentally new discrete IR radiation generation scheme using pulse-width modulation for measuring the dynamics of water vapor and carbon dioxide concentrations, with further application in the instrument base of an ecological and climatic station to implement the eddy covariance (EC) method. In addition, selecting the component base for NGAP-1, as well as its calibration and experimental field validation as part of an ecological and climatic station, are described.

## 1. Introduction

The growing concentrations of key greenhouse gases (GHGs) in the atmosphere—primarily carbon dioxide (${\mathrm{CO}}_{2}$) and methane (${\mathrm{CH}}_{4}$)—are explicitly linked to anthropogenic climate change and dramatic shifts in global biogeochemical cycles [1]. Understanding the sources and sinks of these gases is of critical importance and requires accurate measurement of their net exchange (fluxes) between the atmosphere and various terrestrial and aquatic ecosystems, including forests, wetlands, and agricultural landscapes. These flux measurements provide priceless data for assessing ecosystem responses to environmental variability and management practices, as well as for restricting and testing Earth system models.

The eddy covariance (EC) method has become the standard micrometeorological technique for quantifying fluxes directly at the ecosystem scale over areas ranging from hectares to square kilometers [2,3]. The principle of the method is based on capturing turbulent scalar transport in the near-surface layer of the atmosphere. In particular, the flux (*F*) is calculated as the covariance between high-frequency fluctuations in vertical wind speed (*w*′) and the scalar mixing coefficient or density (*c*′) of interest (i.e., $F=\bar{\left(w^{\prime }c^{\prime }\right)}$), averaged over a suitable period (usually 30–60 min). This direct approach requires instruments capable of detecting these fast turbulent fluctuations. This circumstance entails the use of rapid response (10–20 Hz) and high-precision sensors for determining both the three-dimensional wind speed vector (usually an acoustic anemometer) and the corresponding gas concentrations.

Non-dispersive infrared (NDIR) gas analyzers (IRGAs) have become widely recognized technology for measuring ${\mathrm{CO}}_{2}$ and water vapor ($H_{2}O$) fluxes in the scientific community. Open-path (OP) designs, such as the widely used LI-7500A (LI-COR, Lincoln, NE, USA) and EC150/IRGASON (Campbell Scientific Inc., UT, USA). series instruments, are particularly in demand in many fields. Their advantages include relatively low power consumption, which is crucial for deployment at remote autonomous sites, no signal attenuation or delays associated with the use of samplers, and ease of operation.

However, open-path NDIR analyzers face their own inherent problems. Due to the influence of environmental conditions, they are susceptible to data loss during precipitation, fog, or dew formation on the optical lenses. Flow calculations require significant post-processing to correct for air density variations caused by the simultaneous transfer of heat and water vapor (WPL correction [4]). In addition, past errors associated with device self-heating, especially in low-energy environments, have required complex correction procedures [5]. Even modern optical NDIR designs can exhibit minor deviations related to spectroscopic effects and sensitivity to ambient temperature fluctuations [6,7].

The evolution of NDIR technology has recently bifurcated into two distinct streams. On one hand, high-precision industrial analyzers (e.g., MaxSys 900, HIGNAL TECHNOLOGY Ltd., Shanghai, China) continue to push the boundaries of selectivity using closed-path laser and GFC architectures. On the other, the drive for miniaturization has led to significant advances in MEMS-based sensors for portable applications. Recent works by Xu et al. [8] and Yu et al. [9] have demonstrated the efficacy of MEMS emitters and dual-ellipsoid optical chambers in reducing the footprint and power consumption of handheld gas analyzers. Building upon this solid-state modulation philosophy, our work applies similar MEMS-based PWM principles to the distinct challenge of stationary eddy covariance, where high-frequency response (10 Hz) takes precedence over extreme miniaturization.

Whilst closed-path NDIR analyzers, such as LI-7200RS (LI-COR, Lincoln, NE, USA), were developed to overcome certain operational constraints, they introduce inherent compromises involving frequency response attenuation and sampling tube effects [10]. Devices like LI-7500A use mechanical chopper IR radiation modulation. The use of pulse-width modulation will improve the stability of gas analyzers in field conditions.

Although NDIR is well-established for ${\mathrm{CO}}_{2}$ and $H_{2}O$ analysis, the concurrent monitoring of ${\mathrm{CO}}_{2}$, ${\mathrm{CH}}_{4}$, and $N_{2}O$ via open-path eddy covariance systems typically requires sophisticated power-demanding laser spectroscopy (e.g., TDLAS or QCLS) or the use of several independent analyzers [11,12]. Some locations, e.g., with a limited power supply, require simplicity and reliability of instruments. Thus, this creates limitations for complex monitoring of greenhouse gas fluxes. Here, we present a new open-path analyzer based on NDIR principles, specifically designed for simultaneous high-frequency measurement of ${\mathrm{CO}}_{2}$ and $H_{2}O$ concentrations, suitable for calculating eddy covariance fluxes. The main design principles focused on ensuring reliability in field conditions, optimizing the optical configuration to ensure sensitivity to multiple gases, effective temperature control, and reduced power consumption compared with alternative laser solutions. This article describes in detail the design of the instrument, laboratory performance, and evaluation of field performance.

## 2. Materials and Methods

This section outlines the methodology, where we describe the stages of development of a structurally new NDIR gas analyzer prototype, further mentioned as NGAP-1, Bauman Moscow State Technical University, Moscow, Russia. This instrument is characterized by a fundamentally new, for an open-path fast gas analyzer, discrete IR radiation generation scheme. Unlike LI-7500, NGAP-1 uses pulse-width modulation of the signal to obtain the discrete IR radiation rather than an impeller, which is less reliable and durable. This high-frequency modulation (10 Hz) also effectively filters out the DC component of sunlight and low-frequency ambient radiation changes, ensuring reliable open-path operation without a physical enclosure.

The designed analyzer is intended for integration into complex meteorological stations, specifically for measuring carbon dioxide and water concentration dynamics, especially for the eddy covariance method implementation.

Selecting the component base for the instrument’s development was a crucial step, essential for achieving the requisite high accuracy, sensitivity, and quality of the future product. The main technical components selected for the NGAP-1 gas analyzer include an emitter, a control panel, and matching optics.

### 2.1. IR Emitter

**Figure 1:**
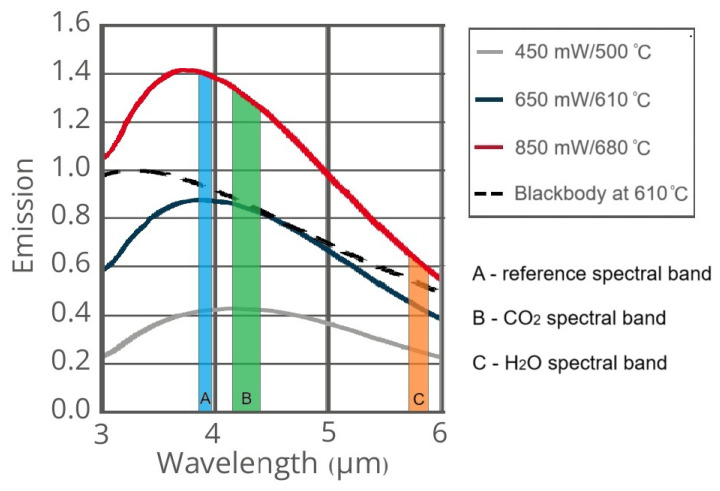
Emission spectrum of the Micro-Hybrid JSIR 350-4 infrared emitter normalized to nominal power and Planck’s Law. The specific spectral bands used NGAP-1 gas analyzer—reference channel, carbon dioxide (${\mathrm{CO}}_{2}$) channel, and water vapor ($H_{2}O$) channel—graphically represented by the blue, green, and orange blocks, respectively.

The Micro-Hybrid JSIR 350-4, Laser Components, Bedford, NH, USA broadband [13] was chosen as the source of IR radiation. The main technical characteristics of the JSIR-350-4 are presented in Table 1 and Figure 1. The instability of the radiation power is determined by the instability of the power sources (no more than 0.1%), which does not significantly contribute to the measured signal.

Where $T_{amb}$ is the ambient temperature. Figure 1 shows JSIR 350-4 emission normalized to nominal power.

### 2.2. Photodetector Device

The InfraTec LRM-244 four-channel pyroelectric photodetector [14] was chosen as the photodetector device. The LRM-244 is highly protected from the effects of external environmental factors due to the silicon central window. The light filters are located inside the photodetector device, which provides protection from environmental influences, including mechanical damage.

The characteristic polarity of the LRM-244 is a unique operational feature, which generates a negative signal when subjected to a positive flow of IR radiation. The characteristics of the LRM-244 channel bandpass filters are shown in Table 2.

The presented photodetector is configured to measure greenhouse gases such as ${\mathrm{CO}}_{2}$ and $H_{2}O$. The characteristics of the bandpass filters of the LRM-244 channels are shown in Table 3.

Channels 2 and 3 are both equipped with bandpass filters at 4.26 μm and 5.80 μm that correspond to the strongest absorption cross-section lines of ${\mathrm{CO}}_{2}$ and $H_{2}O$ according to Hitran database. Channels 1 and 4 are both equipped with a 3.95 μm bandpass filter (reference). We utilized two identical reference channels to implement spatial averaging across the detector array. By averaging the signal from Channels 1 and 4, we reduce the noise level and better compensate for any non-uniformity in the thermal distribution of the source during the PWM heating cycle. Additionally, Channel 4 is used to continuously monitor the radiation intensity of the source to dynamically compensate for aging and drift.

### 2.3. Matching Lenses

Flat-convex Edmund Optics lenses model 87-946 were selected as matching optical elements. These lenses are composed of Burnett’s germanium needle, which has outstanding transmission properties in the IR range. In addition, the lenses have a broadband anti-glare coating for a wavelength range of 3…12 μm, which increases light transmission while reducing surface reflections. The diameter of the 87-946 lens is 25 mm; the focal length is 40 mm.

### 2.4. Developed NGAP-1 Gas Analyzer

**Figure 2:**
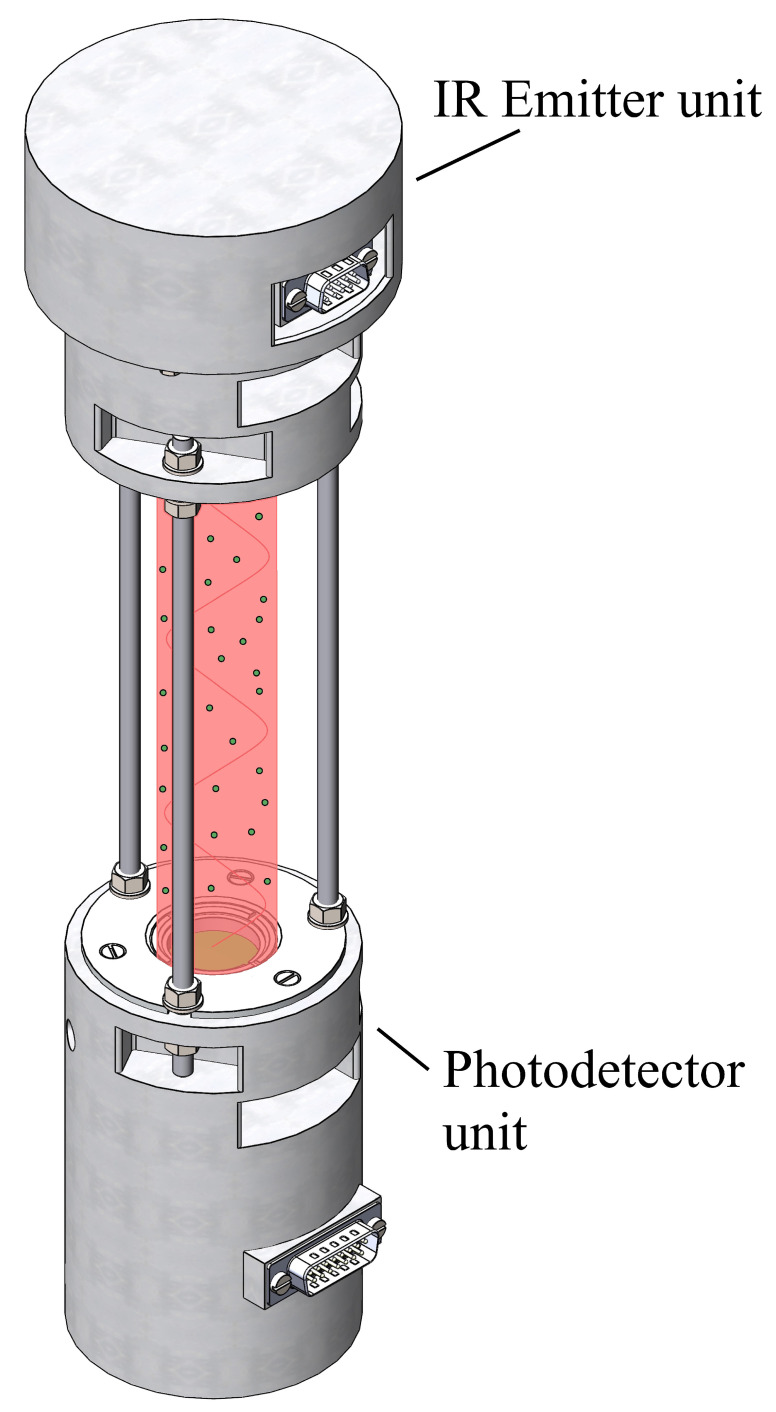
NGAP-1 gas analyzer.

Figure 2 shows overall structure of the instrument. The optical path length, defined as the distance through which the atmospheric air circulates during measurement, is precisely 300 mm. The total device height is 345 mm. The optical design of our open-path probe draws on established principles of non-dispersive infrared sensing, utilizing a dual-wavelength referencing scheme to compensate for source aging and window contamination, a methodology with roots in early double-beam probe designs (e.g., Zhang et al. [15]). NGAP-1 gas analyzer optical path length is 0.15 m in order to measure in photodetector linearity zone. The height of the device is 500 mm.

### 2.5. Calibration

After the device was assembled and the automation software for collecting primary experimental data was developed, the device was calibrated for further field testing as part of an ecological and climatic station and compared with a similar device, LI-7500A, LI-COR, Lincoln, NE, USA.

**Figure 3:**
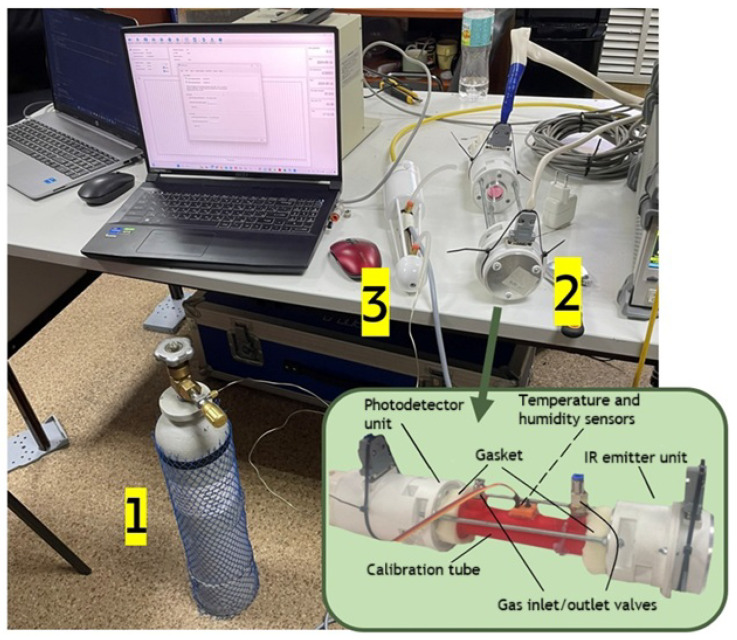
Calibration of NGAP-1 gas analyzer; ${\mathrm{CO}}_{2}$. 1—a gas cylinder with a ${\mathrm{CO}}_{2}$ 600 µmol/mol calibration gas mixture; 2—NGAP-1 gas analyzer with a calibration tube (use only for calibration); 3—LI-7500A with a calibration tube. Experimental conditions: $T_{amb}$ = 21 °C, $T_{dew}$ = 16 °C.

Calibration involved determining a calibration ratio that showed the relation between the values obtained from the photodetector channels and the greenhouse gas concentration. The calibration algorithm for NGAP-1 is described below using the ${\mathrm{CO}}_{2}$ and $H_{2}O$ channels:

(a) A sealed calibration tube with temperature and humidity sensors was placed in the optical path of the NGAP-1 gas analyzer only for the laboratory calibration phase;

(b) Before the calibration with target gases, the values $Z_{{\mathrm{CO}}_{2}}$ and $Z_{H_{2}O}$ were calculated with IR transparent nitrogen ($N_{2}$) blown through the tube;

(c) For carbon dioxide calibration, a ${\mathrm{CO}}_{2}$ calibration gas mixture (600 μmol mol^−1^) was blown through the tube; the values of $Z_{{\mathrm{CO}}_{2}}$ correspond to a concentration of 600 μmol mol^−1^ ${\mathrm{CO}}_{2}$ (Figure 3). For water vapor calibration, conditions close to dew point were set in the calibration tube using a portable dew point generator LI-610, LI-COR, Lincoln, NE, USA. Measurements were conducted at an ambient temperature of 21 °C with a dew point of 16 °C. This resulted in a water vapor concentration of 14.4 mmol/mol for the span calibration of both instruments;

(d) In accordance with the obtained ratios between the concentrations of ${\mathrm{CO}}_{2}$, $H_{2}O$ and $Z_{{\mathrm{CO}}_{2}}$), $Z_{H_{2}O}$, calibration curves were constructed, where $C_{x}$ is the concentration of gas *x* and *A*, *B*, *K*, and *M* are calibration constants (1) and (2):

(1) $$C_{{\mathrm{CO}}_{2}}=A\;\cdot \;Z_{{\mathrm{CO}}_{2}}-B$$

(2) $$C_{H_{2}O}=K\;\cdot \;Z_{H_{2}O}-M$$

The RMS noise of the NGAP-1 gas analyzer for the ${\mathrm{CO}}_{2}$ channel is 0.19 μmol mol^−1^. This value is competitive compared with the claimed sensitivity of 0.10 μmol mol^−1^ ${\mathrm{CO}}_{2}$ for LI-7500A.

### 2.6. Primary Data Processing

**Figure 4:**
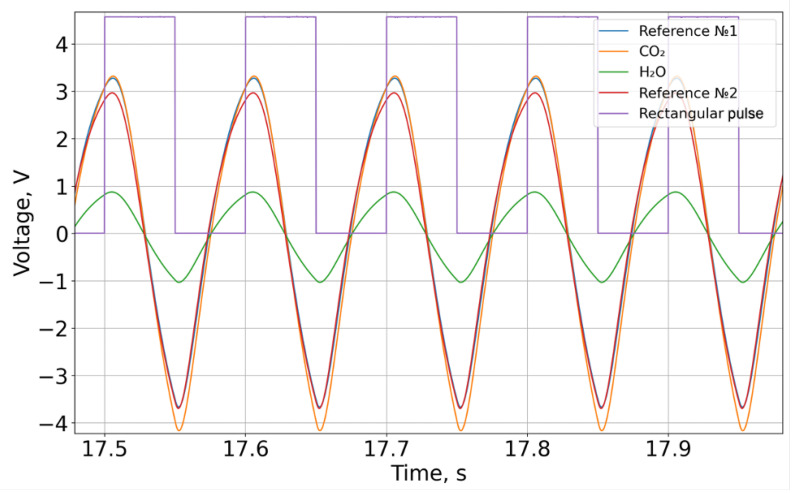
The primary type of signal.

**Figure 5:**
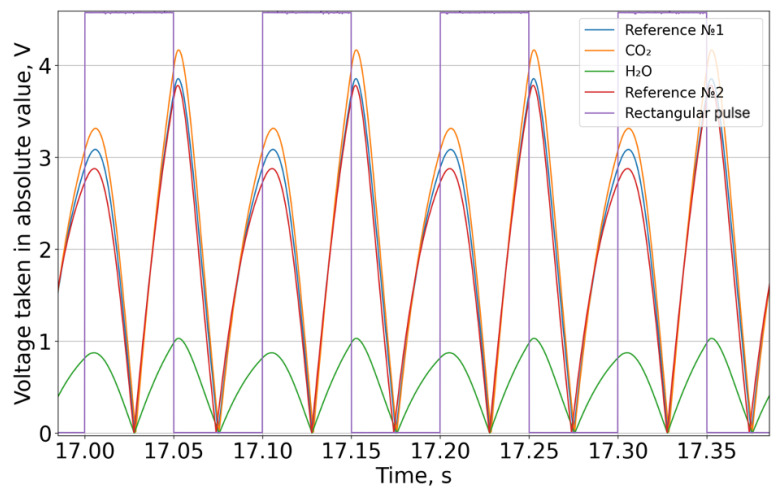
The absolute value of the voltage minus the average value for the period.

**Figure 6:**
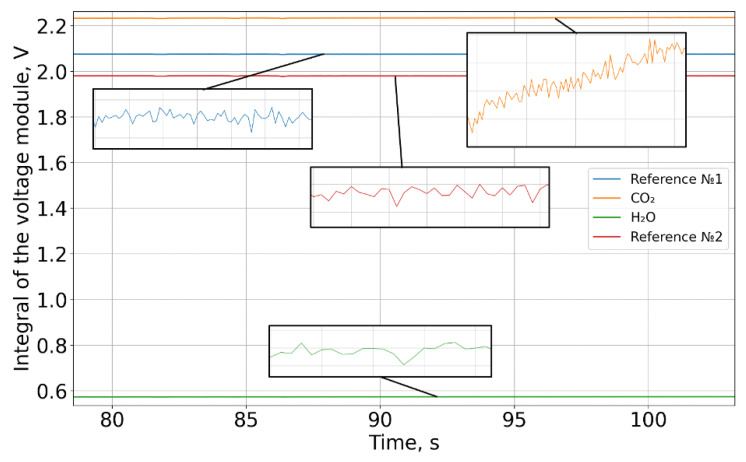
The integral of the absolute voltage value for each period of the rectangular pulse.

A pulse-width modulator generated a periodic control signal with a frequency of 10 Hz, which was fed to a broadband infrared emitter. Each of the four FPA (Focal Plane Array) channels recorded a periodic signal (Figure 4). The data presented in Figure 5 are offset-subtracted and absolute-valued (3). Then, for each of the four channels, the integral over each square wave period was computed and intensity series were constructed (4) (Figure 6).

(3) $$U^{\prime }\left(t\right)=\left|U\left(t\right)-U_{\mathrm{mean}}^{T}\right|,$$

where $U_{\mathrm{mean}}^{T}$ is the mean voltage value over the square wave period.

(4) $$I=\int_{0}^{T}U^{\prime }\left(t\right)\;dt,$$

Next, averaging was performed over two reference channels (5):

(5) $$\langle I_{ref}\rangle =\frac{I_{ref_{1}}+I_{ref_{2}}}{2},$$

where $\langle I_{ref_{1}}\rangle$ and $\langle I_{ref_{2}}\rangle$ are the averaged intensity values of the reference channels.

Then, the quantities proportional to the optical density for carbon dioxide and water vapor were calculated as follows (6) and (7):

(6) $$Z_{{\mathrm{CO}}_{2}}=-\ln\frac{I_{{\mathrm{CO}}_{2}}}{\langle I_{ref}\rangle },$$

(7) $$Z_{H_{2}O}=-\ln\frac{I_{H_{2}O}}{\langle I_{ref}\rangle },$$

where $I_{{\mathrm{CO}}_{2}}$ is the integral for ${\mathrm{CO}}_{2}$ channel; $I_{H_{2}O}$ is the integral for $H_{2}O$ channel.

**Figure 7:**
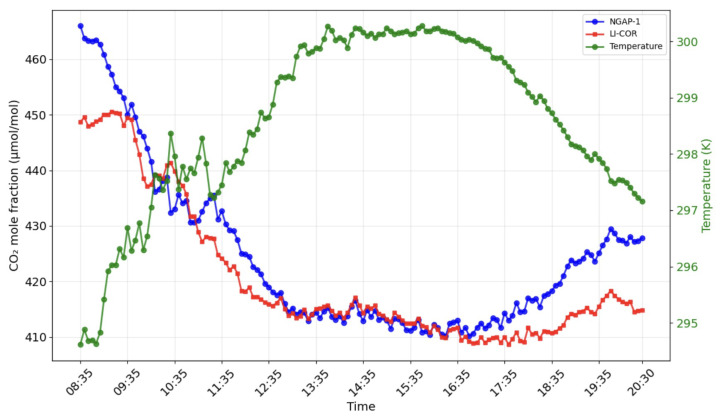
Comparison of ${\mathrm{CO}}_{2}$ mole fraction measurements from LI-COR LI-7500A (red) and NGAP-1 (blue) devices. The green line represents the corresponding air temperature dynamics.

**Figure 8:**
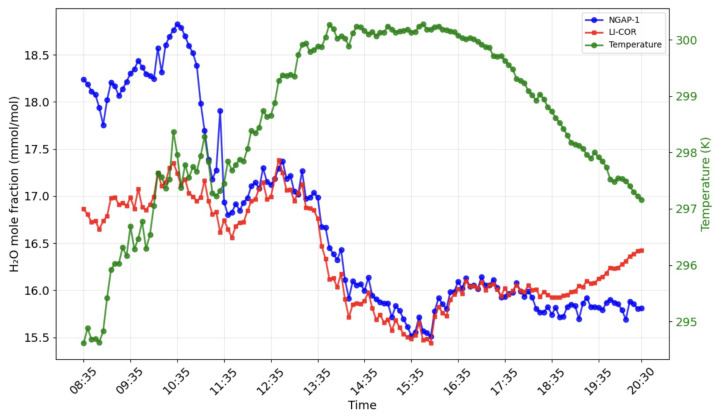
Comparison of $H_{2}O$ mole fraction measurements from LI-COR LI-7500A (red) and NGAP-1 (blue) devices. The green line represents the corresponding air temperature dynamics.

The raw signals from the NGAP-1 gas analyzer were converted into final concentration values by applying the instrument-specific calibration curve (Figure 3). The resulting concentration data are presented in Figure 7 and Figure 8.

### 2.7. Field Verification Site Setup

On 17–18 September and 17 October 2024, a number of experiments were conducted at the Kamshilovka training base of Bauman Moscow State Technical University to test the NGAP-1 gas analyzer and collect and process primary data. The experiments were conducted in order to verify the accuracy of NGAP-1.

**Figure 9:**
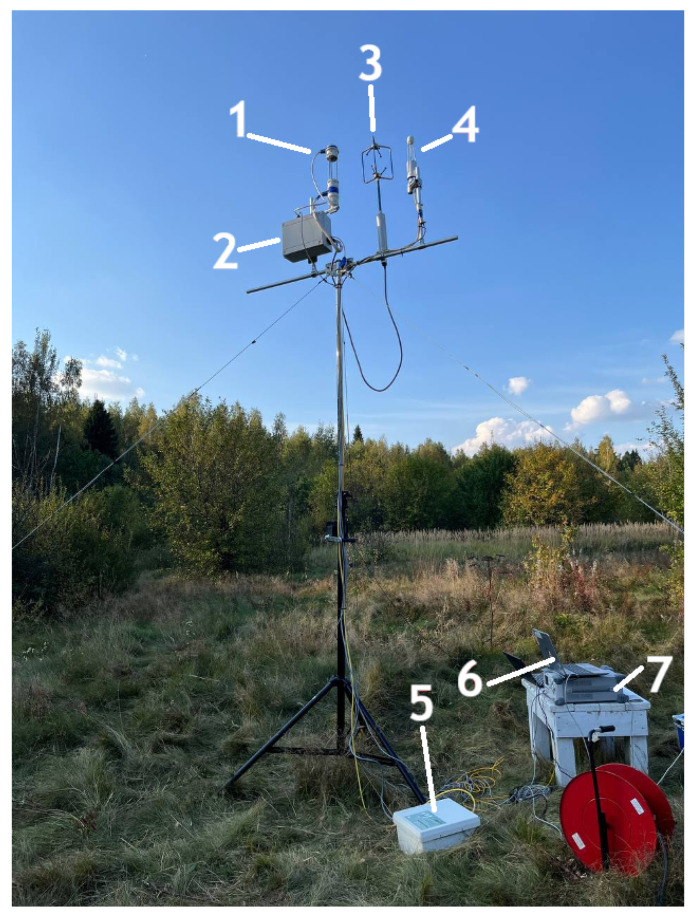
Experimental setup. 1: NGAP-1 gas analyzer; 2: data processing unit for NGAP-1 gas analyzer; 3: ultrasonic anemometer (WindMaster); 4: open-path gas analyzer LI-COR LI-7500A; 5: data processing unit (LI-COR 7550 Interface Unit); 6: control laptop; 7: laboratory power supply.

Figure 9 shows a photograph of the experimental setup, where the devices are placed 3.2 m high above the ground and at a distance of 470 cm from each other.

The integrated measurement system consisted of the following primary components: a four-channel NGAP-1 prototype (NDIR gas analyzer prototype-1) for monitoring water vapor and carbon dioxide; a three-axis WindMaster sonic anemometer (Gill Instruments, Lymington, UK); and a reference LI-7500A open-path gas analyzer (LI-COR, Lincoln, NE, USA. Data acquisition was facilitated by an LI-7550 Analyzer Interface Unit, with a dedicated power supply and communication module supporting the NGAP-1 prototype. High-frequency measurements—specifically ${\mathrm{CO}}_{2}$ and $H_{2}O$ concentrations and the three-dimensional wind components—were recorded at a sampling rate of 10 Hz. Meanwhile, auxiliary meteorological data, including ambient temperature, humidity, and atmospheric pressure, were logged at 1 Hz.

### 2.8. Flux Data Calculation

High-frequency (20 Hz) raw data from the sonic anemometer and infrared gas analyzers were processed using the EddyPro 7.0.9 software (LI-COR Biosciences). Data from NGAP-1 gas analyzer and anemometer data with timestamps were collected to csv files, and, utilizing Python script, converted to ghg files. Python 3.12.9 with Matplotlib 3.10.6, Numpy 2.3.3, and Pandas 2.3.2 libraries were used. Fluxes were computed over 30-min averaging intervals. Prior to covariance calculation, low-frequency trends inherent in the time series were removed using the Block Averaging detrending method. The coordinate system was aligned with the mean wind streamline for each interval using the double rotation method. Any residual tilt in the anemometer’s orientation relative to the mean streamline, not accounted for by double rotation, was addressed using the Planar Fit method [16] applied across a longer dataset encompassing various wind directions. Time lags between the vertical wind component and scalar concentration measurements arising primarily from sensor separation were determined and corrected using the Covariance Maximization method. Fluxes were corrected for density fluctuations induced by concurrent heat and water vapor transport using the Webb–Pearman–Leuning (WPL) correction [4]. While our current prototype is designed for stationary towers, future iterations intended for mobile platforms (buoys and ships) would require additional corrections for platform motion, as detailed by Vandemark et al. [17]. For the terrestrial deployment described herein, static alignment suffices. Data quality assurance was performed using the Foken et al. [18] quality flagging system. A specific correction algorithm (W-boost) designed for Gill WindMaster anemometers was also enabled [19].

## 3. Results

**Figure 10:**
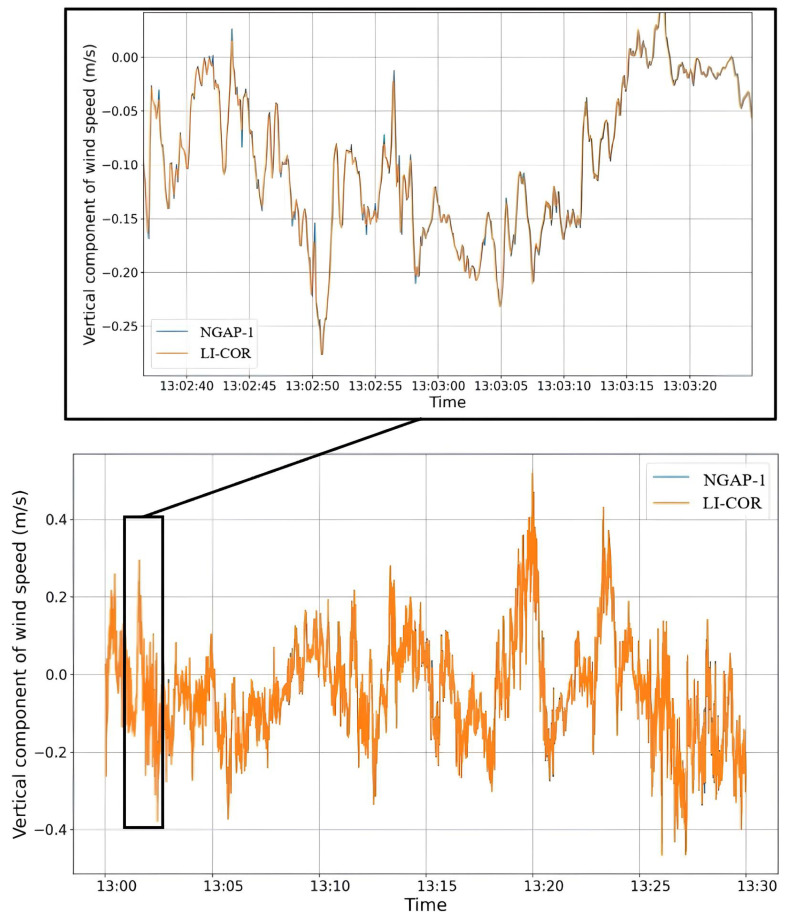
Vertical wind velocity (w) for NGAP-1 (in orange) and LI-7500 (in blue).

Data from the sonic anemometer were transmitted at 10 Hz to the acquisition systems of both gas analyzers simultaneously. This configuration ensured that the measurements from the NGAP-1 prototype and the LI-7500A reference were temporally synchronized using the shared anemometer signal as a common reference (Figure 10).

To accurately calculate carbon dioxide fluxes, it is necessary to consider the effect of dilution with water vapor. An NGAP-1 analyzer measures the mole fraction of ${\mathrm{CO}}_{2}$ relative to humid air (“wet” mole fraction), expressed in μmol mol^−1^. This value is then converted to “dry” mole fraction (i.e., the mole fraction in dry air), as shown in Equation (8):

(8) $$n_{{\mathrm{CO}}_{2_{dry}}}=\frac{n_{{\mathrm{CO}}_{2}}}{1-n_{H_{2}O}}$$

where $n_{{\mathrm{CO}}_{2_{dry}}}$ is the dry mole fraction of ${\mathrm{CO}}_{2}$, $n_{{\mathrm{CO}}_{2}}$ is the mole fraction of ${\mathrm{CO}}_{2}$, and $n_{H_{2}O}$ is the mole fraction of $H_{2}O$.

**Figure 11:**
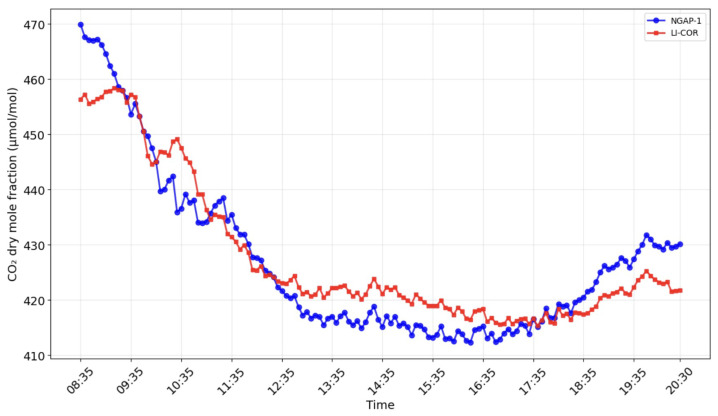
${\mathrm{CO}}_{2}$ dry mole fraction dynamics measured by LI-7500A (red line) and NGAP-1 (blue line) devices.

Figure 11 shows the result of calculating the drained mole fraction of carbon dioxide for NGAP-1 and LI-COR LI-7500A.

**Figure 12:**
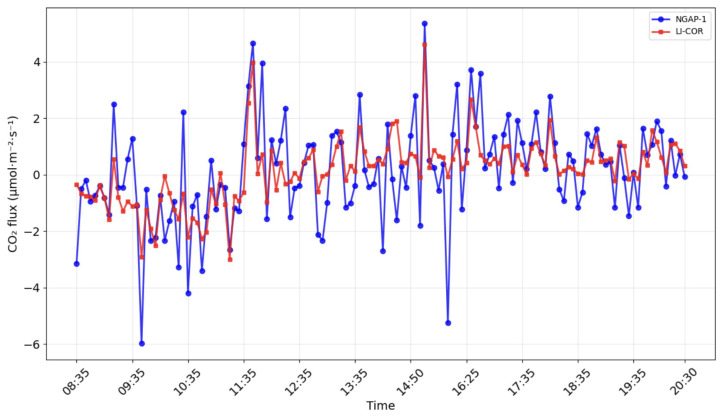
${\mathrm{CO}}_{2}$ flux dynamics calculated with EddyPro software for LI-7500A (red line) and NGAP-1 (blue line) device data.

**Figure 13:**
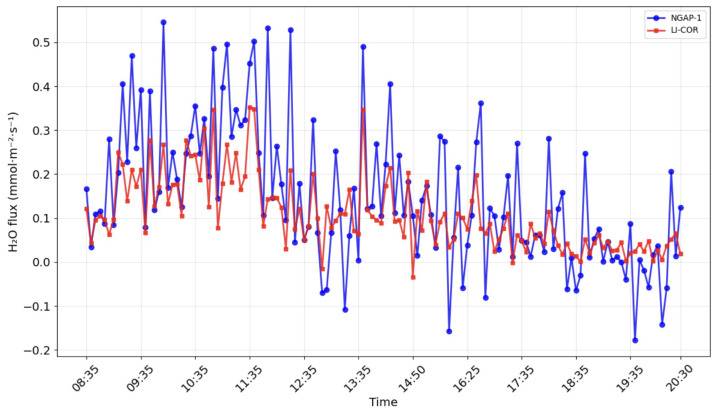
$H_{2}O$ flux dynamics calculated with EddyPro software for LI-7500A (red line) and NGAP-1 (blue line) device data.

Figure 12 and Figure 13 show the results of modeling carbon dioxide and water vapor fluxes based on data obtained from the NGAP-1 gas analyzer and LI-7500A. The simulation was carried out for 12 h (08.30–20.30), conducted on 17.10.24 in Kamshilovka (Moscow Region, Russia). For clarity, a five-minute flux averaging interval was selected.

**Figure 14:**
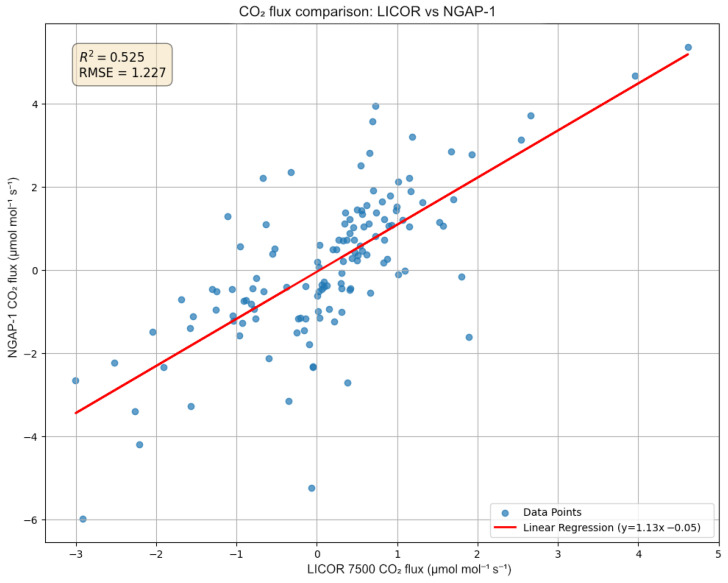
Scatter plot of 5-min ${\mathrm{CO}}_{2}$ fluxes comparing the developed NGAP-1 device (y-axis) against the reference LI-7500A (x-axis). The analysis shows a coefficient of determination ($R^{2}$) of 0.525 and a linear regression of y=1.13x−0.05.

The statistical comparison for the ${\mathrm{CO}}_{2}$ flux (Figure 14) shows a positive correlation, with a coefficient of determination ($R^{2}$) of 0.525 and an RMSE of 1.227 μmol m^−2^s^−1^. While the $R^{2}$ of 0.525 is moderate compared to commercial intercomparisons, it represents a successful validation of the PWM measurement principle as a proof of concept. The linear regression model is retained because the physical relationship between the fluxes is theoretically 1:1. The variance is primarily attributed to the 47 cm sensor separation and the limited duration of the verification period. The linear regression (y=1.13x−0.05) suggests that our NGAP-1 device, in its current calibration state, overestimates the flux magnitude by approximately 13% compared to LI-7500A.

**Figure 15:**
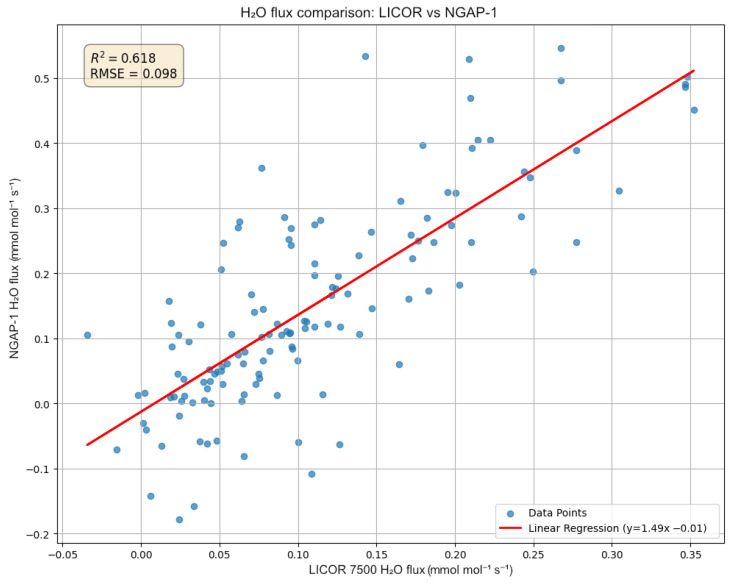
Scatter plot of 5-min $H_{2}O$ fluxes comparing the developed NGAP-1 device (y-axis) against the reference LI-7500A (x-axis). This comparison yielded an $R^{2}$ of 0.618 and a linear regression of y=1.49x−0.01, indicating a significant systematic overestimation by the NGAP-1 gas analyzer.

The comparison for the $H_{2}O$ flux (Figure 15) shows a similar $R^{2}$ of 0.618 with an RMSE of 0.098 mmol m^−2^s^−1^. However, this plot reveals a more significant systematic discrepancy, with a regression slope of 1.49 (y=1.49x−0.01).

These results indicate that, while NGAP-1 successfully captures the diurnal trends (as seen in Figure 12 and Figure 13), the initial linear two-point calibration is not sufficient to fully reconcile the quantitative flux magnitudes between the two instruments. A primary source of the flux magnitude discrepancy is the 47 cm separation distance between NGAP-1 and the reference LI-7500A. At this distance, the instruments are likely sampling different turbulent eddies, particularly at the high frequencies required for eddy covariance.

## 4. Discussion

The landscape of ${\mathrm{CO}}_{2}$ monitoring has been broadened by the availability of low-cost sensors such as the K30 (SenseAir, Delsbo, Sweden). As evaluated by Martin et al. [20], these sensors offer a viable pathway for spatially dense monitoring networks where high temporal resolution is secondary. However, their diffusion-limited response times render them unsuitable for direct eddy covariance flux measurements, which require resolving turbulent eddies in the 5–10 Hz range. NGAP-1 bridges this gap, offering the requisite speed of research-grade analyzers with a simplified chopper-less architecture.

The paper presents the results of the development and field validation of an open-path NDIR gas analyzer, NGAP-1, designed to measure water and carbon fluxes using the eddy covariance method.

The primary objective was to create a field prototype of the fast gas analyzer with increased reliability and lower cost by eliminating mechanical signal modulation components. As an innovative solution, the mechanical chopper was replaced with a solid-state pulse-width modulation (PWM) circuit for the infrared emitter. This approach improves manufacturability by reducing the number of components, eliminates the risk of electric motor failure, and reduces cost, which is crucial when creating a prototype for mass production and long-term operation in remote and extreme conditions. Moreover, it is possible to change the modulation frequency using software for different tasks, which does not require reworking the chopper design and makes the described design the most versatile.

Field verification confirmed the operability of the proposed design. The key quality criterion for an eddy covariance analyzer is its ability to detect high-frequency fluctuations in turbulent flows, and, in this respect, the NGAP-1 prototype has demonstrated satisfactory results. A comparison of the data showed comparability of fluxes reflecting daily absorption by the ecosystem and evapotranspiration for NGAP-1 and the reference device LI-7500A (Figure 12 and Figure 13). The observed diurnal variation in ${\mathrm{CO}}_{2}$ concentration (>50 μmol mol^−1^) is consistent with biologically active rural sites, where nocturnal respiration accumulates in the stable boundary layer and daytime photosynthesis draws concentrations down.

It was established that a sampling frequency of 10 Hz combined with the implemented signal processing algorithm is sufficient for eddy covariance measurements.

The spectral lines of the main atmospheric gas components do not strongly interfere with the $H_{2}O$ and ${\mathrm{CO}}_{2}$ lines. There is some overlap between the ${\mathrm{CO}}_{2}$ and CO lines, but the difference in concentration by several orders of magnitude makes it possible to neglect the effect of absorption of other atmospheric gases.

It is important to discuss the instrument’s performance specifications in context. Our laboratory calibration determined an RMS noise at 600 μmol mol^−1^ ${\mathrm{CO}}_{2}$ and at 10 Hz of 0.19 μmol mol^−1^. For LI-7500A, the RMS noise at 370 μmol mol^−1^ ${\mathrm{CO}}_{2}$ and at 10 Hz is 0.10 μmol mol^−1^. The field results clearly indicate that this level of precision is more than adequate to resolve eddy covariance. The tight tracking of fluxes that are dependent on resolving small rapid changes is a more practical validation of the instrument’s performance than a static sensitivity metric alone. The accuracy of the obtained data was further confirmed by the successful application of the standard WPL correction, indicating their compatibility with generally accepted methods for processing the eddy covariance measurement results.

We strongly believe that the differences in LI-7500A and NGAP-1 readings obtained in the morning and evening (Figure 7 and Figure 8) are primarily attributed to the ’self-heating’ or ’instrument surface heating’ effect, a well-documented phenomenon in open-path gas analyzers. The core principle of the correction proposed by Burba et al. [5] is that heat dissipated by an instrument’s electronics and optical surfaces can warm the air within the measurement path. This localized heating reduces the air density relative to the surrounding environment. Because NDIR analyzers measure gas concentrations based on density, this thermal expansion leads to a systematic underestimation of the gas concentration if only the ambient temperature is considered. While the ’Burba correction’ is widely used to mitigate these errors for LI-7500A, it is a semi-empirical algorithm that is highly dependent on the specific geometry and thermal properties of the instrument’s chassis. As NGAP-1 is a prototype with a unique physical design, the existing corrections for the LI-7500A device cannot be directly applied. We are currently developing a final version of the instrument with a redesigned body; once this hardware is finalized, we intend to conduct dedicated laboratory and field experiments to characterize its specific thermal response and implement a bespoke correction algorithm. At this stage of development, the figures reflect the raw performance of the prototype relative to the reference instrument.

Regarding temperature effects, the dual-wavelength ratio method (signal/reference) inherently compensates for thermal drift affecting the source and detector. Furthermore, the Ideal Gas Law is applied in post-processing to correct for air density changes due to ambient temperature fluctuations, ensuring accurate mixing ratio calculations without the strict need for physical recalibration at every temperature. Measurement errors are often determined by the power supply circuit of the photodetector and the analog-to-digital converter and can be improved upon transition to mass production.

The authors are aware that the current version of the device and the verification procedure have a number of shortcomings. For example, the field experiment was conducted for over a 12-h period under favorable meteorological conditions. This work did not evaluate the long-term stability of the device, possible drifts in readings, or data loss under unfavorable weather conditions—in particular during rain, fog, and dew formation. All of this is planned to be carried out in the next version of the device with a housing designed for field testing. A separate comparative analysis of the energy consumption of the designed analyzer with systems using mechanical modulators is planned.

## 5. Conclusions

This work successfully detailed the development stages of an open-path fast NDIR gas analyzer featuring a pulse-width modulation IR radiation generation scheme. The primary achievement is the creation of an instrument specifically designed to dynamically measure water vapor and carbon dioxide concentrations. Crucially, the gas analyzer was developed with the explicit purpose of integration into ecological and climatic stations to enable the application of the eddy covariance method for ${\mathrm{CO}}_{2}$ and $H_{2}O$ flux measurements. The systematic process of selecting critical components for the NGAP-1 system was completed, and the essential steps of calibrating the instrument were undertaken. The RMS noise at 600 μmol mol^−1^ ${\mathrm{CO}}_{2}$ and at 10 Hz modulation was 0.19 μmol mol^−1^. Finally, the successful execution of field experiments, conducted as part of an ecological and climatic station deployment, demonstrates the practical implementation and competitiveness of NGAP-1 for its intended environmental monitoring role. We used NGAP-1 and Li-Cor 7500A in the same experimental setup. This combined system made it possible to compare measurement results in real time and compare with an industry standard such as Li-Cor 7500A.

This work serves as a successful proof of concept. The clear next steps are to advance from this prototype to a field-ready version for long-term deployment. Future work will focus on quantifying long-term stability, data retention during adverse weather, and conducting a detailed power consumption analysis. This positions the instrument as a promising, reliable, and cost-effective alternative for future ecosystem-scale flux monitoring.

## Figures and Tables

**Table 1:** Technical characteristics of JSIR 350-4.

Parameter	Value	Units	Additional Notes
Spectral range	2…15	μm	–
Active area	2.2 × 2.2	$mm^{2}$	–
Thermal resistance (*R*)	40 ± 20	Ω	At nominal power
Temperature coefficient	500	μmol·mol^−1^·K^−1^	At 25–800 °C
Time constant	12.5	ms	–
Nominal power consumption	650	mW	In on state
Operating voltage	4.9	V	At R = 40 Ω
Operating current	132	mA	At R = 40 Ω
Active area temperature (*T*)	610 ± 30	°C	At nominal power; $T_{\mathrm{amb}}$ = 25 °C
Estimated service life	>5000	h	740 °C ($T+T_{\mathrm{amb}}$)
	>100,000		610 °C ($T+T_{\mathrm{amb}}$)
**Limiting values of some parameters**			
Input power	1200	mW	In on state; $T_{\mathrm{amb}}$ = 25 °C
Case temperature	120	°C	$T+T_{\mathrm{amb}}$
*T*	850	°C	–

**Table 2:** Technical characteristics of LRM-244.

Parameter	Value	Units
Aperture size	8.5 × 8.5	mm^2^
Photocell size	2 × 2	mm^2^
Thermal time constant	150	ms
Feedback resistor	100 ± 20%	GΩ
Feedback capacitor	0.2 ± 0.1	pF
Voltage sensitivity	80,000	V W^−1^
Maximum noise density	55	$\mu V/\sqrt{\mathrm{Hz}}$
Detectivity	$6\times 10^{8}$	cm·$\sqrt{\mathrm{Hz}}$/W
Supply voltage V+/V−	16	V
Operating supply voltage V+/V−	+2.2…8.0/−2.2…−8.0	V
Recommended supply voltage V+/V−	+5/−5	V
Maximum current consumption	150	μA
Offset voltage	−5…+5	mV
Optimal load	470	kΩ
Maximum output current	±0.4	mA
Operating temperature	−25…+85	°C

**Table 3:** Technical characteristics of bandpass filters of LRM-244 channels.

Parameter	Channel 1	Channel 2	Channel 3	Channel 4
Channel assignment	Reference 1	CO_2_	H_2_O	Reference 2
Bandpass filter, nm	3950	4260	5800	3950
Filter bandwidth at half height, nm	90	180	100	90

## Data Availability

The data presented in this study are available on request from the corresponding author. The data are not publicly available because of privacy restrictions.
